# [^225^Ac]Ac- and [^111^In]In-DOTA-trastuzumab theranostic pair: cellular dosimetry and cytotoxicity in vitro and tumour and normal tissue uptake in vivo in NRG mice with HER2-positive human breast cancer xenografts

**DOI:** 10.1186/s41181-023-00208-0

**Published:** 2023-09-26

**Authors:** Misaki Kondo, Zhongli Cai, Conrad Chan, Nubaira Forkan, Raymond M. Reilly

**Affiliations:** 1 Department of Pharmaceutical Sciences, Leslie Dan Faculty of Pharmacy, University of Toronto, Toronto, ON M5S 3M2 Canada; 2 Princess Margaret Cancer Centre, Toronto, ON Canada; 3 Department of Medical Imaging, University of Toronto, Toronto, ON Canada; 4 Joint Department of Medical Imaging, University Health Network, Toronto, ON Canada

**Keywords:** Trastuzumab, ^225^Ac, α-particles, HER2, Breast cancer, ^111^In

## Abstract

**Background:**

Trastuzumab (Herceptin) has improved the outcome for patients with HER2-positive breast cancer (BC) but brain metastases (BM) remain a challenge due to poor uptake of trastuzumab into the brain. Radioimmunotherapy (RIT) with trastuzumab labeled with α-particle emitting, ^225^Ac may overcome this challenge by increasing the cytotoxic potency on HER2-positive BC cells. Our first aim was to synthesize and characterize [^111^In]In-DOTA-trastuzumab and [^225^Ac]Ac-DOTA-trastuzumab as a theranostic pair for imaging and RIT of HER2-positive BC, respectively. A second aim was to estimate the cellular dosimetry of [^225^Ac]Ac-DOTA-trastuzumab and determine its cytotoxicity in vitro on HER2-positive BC cells. A third aim was to study the tumour and normal tissue uptake of [^225^Ac]Ac-DOTA-trastuzumab using [^111^In]In-DOTA-trastuzumab as a radiotracer in vivo in NRG mice with s.c. 164/8-1B/H2N.luc^+^ human BC tumours that metastasize to the brain.

**Results:**

Trastuzumab was conjugated to 12.7 ± 1.2 DOTA chelators and labeled with ^111^In or ^225^Ac. [^111^In]In-DOTA-trastuzumab exhibited high affinity specific binding to HER2-positive SK-BR-3 human BC cells (K_D_ = 1.2 ± 0.3 × 10^–8^ mol/L). Treatment with [^225^Ac]Ac-DOTA-trastuzumab decreased the surviving fraction (SF) of SK-BR-3 cells dependent on the specific activity (SA) with SF < 0.001 at SA = 0.74 kBq/µg. No surviving colonies were noted at SA = 1.10 kBq/µg or 1.665 kBq/µg. Multiple DNA double-strand breaks (DSBs) were detected in SK-BR-3 cells exposed to [^225^Ac]Ac-DOTA-trastuzumab by γ-H2AX immunofluorescence microscopy. The time-integrated activity of [^111^In]In-DOTA-trastuzumab in SK-BR-3 cells was measured and used to estimate the absorbed doses from [^225^Ac]Ac-DOTA-trastuzumab by Monte Carlo N-Particle simulation for correlation with the SF. The dose required to decrease the SF of SK-BR-3 cells to 0.10 (D_10_) was 1.10 Gy. Based on the D_10_ reported for γ-irradiation of SK-BR-3 cells, we estimate that the relative biological effectiveness of the α-particles emitted by ^225^Ac is 4.4. Biodistribution studies in NRG mice with s.c. 164/8-1B/H2N.luc^+^ human BC tumours at 48 h post-coinjection of [^111^In]In-DOTA-trastuzumab and [^225^Ac]Ac-DOTA-trastuzumab revealed HER2-specific tumour uptake (10.6 ± 0.6% ID/g) but spleen uptake was high (28.9 ± 7.4% ID/g). Tumours were well-visualized by SPECT/CT imaging using [^111^In]In-DOTA-trastuzumab.

**Conclusion:**

We conclude that [^225^Ac]Ac-DOTA-trastuzumab exhibited potent and HER2-specific cytotoxicity on SK-BR-3 cells in vitro and HER2-specific uptake in s.c. 164/8-1B/H2N.luc^+^ human BC tumours in NRG mice, and these tumours were imaged by SPECT/CT with [^111^In]In-DOTA-trastuzumab. These results are promising for combining [^111^In]In-DOTA-trastuzumab and [^225^Ac]Ac-DOTA-trastuzumab as a theranostic pair for imaging and RIT of HER2-positive BC.

**Supplementary Information:**

The online version contains supplementary material available at 10.1186/s41181-023-00208-0.

## Background

There is great interest in treating cancer with biomolecules including monoclonal antibodies (mAbs) and their fragments or peptides labeled with α-particle emitting radionuclides targeted to receptors overexpressed on tumour cells (Aghevlian et al. 2017; Morgenstern et al. 2018). Actinium-225 (^225^Ac; t_1/2_ = 10 d) decays by α-particle emission (Eα = 5.8 MeV) to stable ^209^Bi through a series of daughter radionuclides that emit α-particles (^221^Fr, ^211^At, ^213^Bi, ^213^Po) or β-particles (^213^Bi, ^209^Tl, ^209^Pb) (Aghevlian et al. 2017). An attractive property of α-particles, which makes them potent for cancer treatment is their high linear energy transfer (LET = 50–230 keV/µm), since several MeV of α-particle energy are deposited over a range of only 28–100 µm (~ 3–10 cell diameters). In contrast, the β-particles emitted by ^177^Lu (Eβ_max_ = 0.5 MeV, t_1/2_ = 6.7 d), which has been more widley used for cancer treatment, deposit their energy over a distance up to 1.8 mm (~ 180 cell diameters), resulting in very low LET < 0.1 keV/µm, and consequently lower cytotoxic potency. Furthermore, α-particles directly inflict lethal DNA double-strand breaks (DSBs) in cancer cells, in addition to causing indirect DNA damage mediated by reactive oxygen species (ROS), while β-particles rely on ROS-mediated DNA damage, making treatment with α-particles less susceptible to tumour hypoxia (Seidl 2014; Wulbrand et al. 2013). Thus, α-particles are more powerful and more precise for killing cancer cells than β-particles and may be able to overcome resistance to other types of radiation used for targeted radiotherapy of cancer. Indeed, a patient with widespread metastatic prostate cancer resistant to treatment with β-particle emitting, ^177^Lu-labeled PSMA-617, a radiopeptide targeted to prostate specific membrane antigen (PSMA), achieved a complete remission when treated with α-particle emitting, ^225^Ac-labeled PSMA-617 with disappearance of tumours on positron-emission tomography (PET) scans using [^68^ Ga]Ga-PSMA11 and serum prostate specific antigen (PSA) levels decreasing almost 3000-fold to < 0.1 ng/mL (Kratochwil et al. 2016).

Radioimmunotherapy (RIT) with mAbs labeled with α-particle emitters has been studied with encouraging results across several different cancer types (Aghevlian et al. 2017; Seidl 2014). Our group aims to study α-particle RIT of human epidermal growth factor receptor-2 (HER2)-positive breast cancer (BC), particularly treatment of brain metastases (BM) by delivering trastuzumab (Herceptin) linked to 1,4,7,10-tetraazacyclododecane-1,4,7,10-tetraacetic acid (DOTA) complexed to ^225^Ac ([^225^Ac]Ac-DOTA-trastuzumab) to the brain. HER2-positive BC is a subtype of BC that accounts for 10–15% of cases (Burstein 2005). Overexpression of HER2 confers an aggressive phenotype and patients with this subtype of BC previously had a poor prognosis (Slamon et al. 1987). However, the introduction of HER2-targeted mAb therapies including trastuzumab (Herceptin, Roche), pertuzumab (Perjeta, Roche), trastuzumab-emtansine (Kadcyla, Roche) and the HER2 tyrosine kinase inhibitors lapatinib (Tykerb, GlaxoSmithKline) and tucatinib (Tukysa, Cascadian Therapeutics) has improved the outcome of patients with HER2-positive BC (Swain et al. 2023). Nonetheless, there remain important challenges including resistance to HER2-targeted therapies (Elshazly and Gewirtz 2022) and low uptake of mAb-based agents into metastases in the brain due to poor penetration across the blood–brain-barrier (BBB) (Bailleux et al. 2021).

Since radiation kills cancer cells by a mechanism that is independent of inhibition of the growth promoting function of HER2, RIT may overcome resistance to these therapies. Our group reported that trastuzumab modified with nuclear translocation sequence (NLS) peptides and modified with diethylenetriaminepentaacetic acid (DTPA) complexed to the Auger electron-emitting ^111^In ([^111^In]In-DTPA-trastuzumab-NLS) was more effective than trastuzumab for treatment of human HER2-positive BC tumours in mice (Costantini et al. 2010) and killed HER2-positive BC cells in vitro that were resistant to trastuzumab (Costantini et al. 2008). Auger electrons are analogous to α-particles in that they are a high LET form of radiation (LET = 4–26 keV/µm), although these electrons have much lower energy (< 20 keV) and a subcellular range (nanometers to micrometers) (Aghevlian et al. 2017; Ku et al. 2019). The powerful cytotoxic properties of α-particles emitted by [^225^Ac]Ac-DOTA-trastuzumab may make this agent more effective than trastuzumab for treatment of HER2-positive BC, particularly when delivered to the brain, and may overcome resistance of these tumours to HER2-targeted agents. Two strategies to improve the uptake of [^225^Ac]Ac-DOTA-trastuzumab in BM are: i) MRI-guided focused ultrasound (MRIg-FUS) which transiently and spatially disrupts the BBB around BM to enhance the delivery of intravenously (i.v.) injected radioimmunoconjugates (RICs) and ii) convection-enhanced delivery (CED) in which RICs are infused directly into BM using a catheter. We recently reported that MRIg-FUS increased the uptake of i.v. injected [^111^In]In-DTPA-trastuzumab in BM in patients with HER2-positive BC by up to fourfold (Meng et al. 2021). Radiolabeled trastuzumab has not been delivered to BM in patients by CED, but trastuzumab infused into HER2-positive human BC tumours in the brain in athymic rats increased survival by twofold compared to intraperitoneally (i.p.) administered trastuzumab, suggesting CED improved delivery to these tumours (Grossi et al. 2003). The safety of CED delivery of monoclonal antibodies to tumours in the brain is shown by a Phase I study of ^124^I-labeled 8H9 antibodies administered by CED in patients with diffuse intrinsic pontine glioma, which found no dose-limiting toxicity (Souweidane et al. 2018). However, the safety of CED for delivery of [^225^Ac]Ac-DOTA-trastuzumab to BM in patients with HER2-positive BC remains to be evaluated.

Our objectives in this study were to: (i) synthesize and characterize [^225^Ac]Ac-DOTA-trastuzumab, (ii) determine its ability to decrease the clonogenic survival fraction (SF) of HER2-positive SK-BR-3 human BC cells in vitro compared to trastuzumab and measure DNA DSBs caused by ^225^Ac and its daughters, (iii) estimate the cellular dosimetry of [^225^Ac]Ac-DOTA-trastuzumab and correlate the SF with the radiation absorbed dose in SK-BR-3 cells, and (iv) assess its tumour and normal tissue uptake in vivo in NOD-*Rag1*^*null*^* IL2rg*^*null*^ (NRG) mice with subcutaneous (s.c.) HER2-positive 164/8-1B/H2N.luc^+^ human BC xenografts. There have been a few previously reported preclinical studies of ^225^Ac-labeled trastuzumab for treatment of HER2-positive BC (Ballangrud et al. 2004; Borchardt et al. 2003; Yoshida et al. 2016), but none have reported the absorbed doses in HER2-positive SK-BR-3 BC cells or determined the relationship between dose and the SF in clonogenic survival assays, or measured DNA DSBs caused by emission of α-particles. Moreover, 164/8-1B/H2N.luc^+^ cells were selected here for tumour and normal tissue uptake studies, because tumour xenografts established from these cells in immunocompromised mice have been shown to have a high affinity for metastasis to the brain (Milsom et al. 2013). This will enable our future studies of [^225^Ac]Ac-DOTA-trastuzumab for RIT of BM from HER2-positive BC in mouse tumour xenograft models.

## Methods

### Cell culture and tumour xenograft mouse model

SK-BR-3 human BC cells overexpressing HER2 (1.3 × 10^6^ receptors/cell (McLarty et al. 2009)) were purchased from the American Type Culture Collection (Manassas, VA). 164/8-1B/H2N.luc^+^ human BC cells derived from MDA-MB-231 human BC cells with metastatic affinity for the brain (Milsom et al. 2013) that overexpress HER2 were donated by Dr. Robert S. Kerbel, Sunnybrook Health Sciences Centre. SK-BR-3 and 164/8-1B/H2N.luc^+^ cells were cultured in RPMI 1640 medium (Sigma-Aldrich, St. Louis, MO, USA) supplemented with 10% fetal bovine serum (FBS; Invitrogen, Carlsbad, CA, USA) at 5% CO_2_/37 °C. Human BC xenografts were established in female NRG 6–8 week old mice by s.c. inoculation of 4.8 × 10^5^ 164/8-1B/H2N.luc + cells in 200 μL of a 1:1 mixture of Matrigel (BD Biosciences, Franklin Lakes, NJ) and serum-free media. Tumours (4–9 mm diameter) were formed after 10 d.

## Synthesis and characterization of immunoconjugates

Trastuzumab (Herceptin®; Hoffman La Roche, Mississauga, ON, Canada) was purchased from the Princess Margaret Cancer Centre (Toronto, ON, Canada) pharmacy and reconstituted with the supplied Bacteriostatic Water for Injection, USP to 21 mg/mL following the manufacturer's directions. Reconstituted trastuzumab was buffer-exchanged into 0.1 M NaHCO_3_ buffer, pH 8.2 at room temperature (RT) on an Amicon Ultra 0.5 ultracentrifugal filtration device (30 kDa MW cut-off) and the protein concentration measured by determining the absorbance at 280 nm (A_280_ of a 1 mg/mL solution = 1.47). Buffer-exchanged trastuzumab (15–20 mg/mL) was then conjugated to 1,4,7,10-tetraazacyclododecane-1,4,7,10-tetraacetic acid (DOTA) by reaction with a 10-, 30 or 60-fold molar excess of DOTA N-hydroxysuccinimide ester (DOTA-NHS; Macrocyclics, Dallas, TX) for 2 h at RT. DOTA-trastuzumab was purified and buffer-exchanged into 0.1M NH_4_Ac buffer, pH 5.5 by transferring the reaction mixture to an Amicon Ultra 0.5 ultracentrifugal device, diluting with 0.1M NH_4_Ac buffer, pH 5.5 to a volume of 500 µL and centrifuging at 7000×*g* for 5 min. The retentate was rediluted with 300 µL of 0.1M NH_4_Ac buffer, pH 5.5 and ultracentrifugation was repeated a total of 6 times. Purified DOTA-trastuzumab was recovered and the protein concentration measured and adjusted to 20–30 mg/mL with 0.1M NH_4_Ac buffer, pH 5.5. Irrelevant human IgG_1_ (Sigma-Aldrich Product No. 15154) was conjugated to DOTA by reaction of 5 mg (200 µL) in 0.1 M NaHCO_3_ buffer, pH 8.2 with a 30-fold molar excess of DOTA-NHS (Macrocyclics) for 2 h at RT, and purifying the DOTA-IgG_1_ immunoconjugates on an Amicon Ultra 0.5 ultracentrifugal device (Amicon).

The purity and homogeneity of DOTA-trastuzumab was assessed by sodium dodecyl sulfate polyacrylamide gel electrophoresis (SDS-PAGE) on a 4–20% Mini-Protean Tris/glycine mini-gel (Bio-Rad, Hercules, CA, USA). The bands were stained with Coomassie Brilliant Blue G-250 (BioRad). Broad range MW markers (BLUeye Prestained Protein Ladder, FroggaBio, Concord, ON, Canada) were electrophoresed to calibrate the gel. In addition, size-exclusion HPLC (SE-HPLC) was performed on a BioSep SEC-S2000 column (Phenomenex, Torrance, CA, USA) eluted with 0.1 M NaH_2_PO_4_ buffer, pH 7.0 at a flow rate of 0.8 mL/min with UV detection at 280 nm. The number of DOTA conjugated per trastuzumab molecule after reaction with a 30-fold excess of DOTA-NHS was determined by analysing the mass spectra of trastuzumab and DOTA-trastuzumab by matrix-assisted laser desorption ionization time-of-flight mass spectrometry (MALDI-TOF MS) at the AIMS Mass Spectrometry Laboratory in the Department of Chemistry, University of Toronto (Toronto, ON, Canada). The mass difference for DOTA-trastuzumab compared to trastuzumab determined by the *m/z* value of the single-protonated (1^+^ charge state) mass spectral peaks was divided by the mass of DOTA (387.41 mass units) to calculate the number of DOTA conjugated to trastuzumab. The number of DOTA conjugated per trastuzumab was determined for reaction of trastuzumab with a 10-fold, 30-fold and 60-fold excess of DOTA-NHS by measuring the conjugation efficiency (CE) following trace labeling with ^111^In by determining the proportion of [^111^In]In-DOTA-trastuzumab and unconjugated [^111^In]In-DOTA by instant thin layer-silica gel chromatography (ITLC-SG) developed in 0.1 M sodium citrate buffer, pH 5.5 as described in the next section. This CE was multiplied by the molar ratio for the reaction to calculate the number of DOTA per trastuzumab.

## Labeling of immunoconjugates with ^111^In and ^225^Ac

DOTA-trastuzumab was labeled with ^111^In by incubation of 100 µg (4 µL in 0.1M NH_4_Ac buffer, pH 5.5) with [^111^In]InCl_3_ (BWXT Medical Ltd, Ottawa, ON, Canada) pre-mixed with an equal volume of 0.1M NH_4_Ac buffer, pH 5.5. Conditions were studied for ^111^In labeling of DOTA-trastuzumab including temperature (25, 32, 37 or 40 °C), incubation time (0.5, 1, 1.5, 2, 3 h), specific activity (SA = 0.111, 0.185, 0.26 MBq/µg) and protein concentration (2.5, 5.0, 7.5, 10.0, 20.0 mg/mL). The SA of ^[111^In]In-DOTA-trastuzumab was based on the amount of ^111^In incubated with the immunoconjugates since no post-labeling purification was required to achieve high radiochemical purity (RCP > 90%). The RCP of [^111^In]In-DOTA-trastuzumab was measured by instant thin layer silica gel chromatography (ITLC-SG; Agilent Technologies, Santa Clara, CA, USA) developed in 0.1 M sodium citrate buffer, pH 5.5. The R_f_ values of [^111^In]In-DOTA-trastuzumab and [^111^In]In-DOTA or free ^111^In were 0.0 and 1.0, respectively. DOTA-trastuzumab was labeled with ^225^Ac following the protocol described by Maguire et al. (2014). ^225^Ac was supplied by the U.S. Department of Energy Isotope Program (Oak Ridge, TN, USA), managed by the Office of Isotope R&D and Production. First, 37 MBq of dry [^225^Ac]Ac(NO_3_)_3_ was dissolved in 10 µL 0.2 M Optima grade high purity HCl (Thermo Fisher Scientific, Waltham, MA, USA). ^225^Ac (1.5 MBq) in 0.2 M HCl measured by a dose calibrator was incubated with 2 M tetramethylammonium acetate buffer (25 µL), L-ascorbic acid (150 g/L; 10 µL), and DOTA-trastuzumab (100 µg; 3.0 µL) in 0.1M NH_4_Ac buffer, pH 5.5. Post-labeling purification was performed by ultrafiltration on an Amicon Ultra 0.5 ultracentrifugal unit (30 kDa MW cut-off) centrifuged at 7000×*g* and recovering the retentate containing [^225^Ac]Ac-DOTA-trastuzumab. The SA of [^225^Ac]Ac-DOTA-trastuzumab was measured after post-labeling purification and ranged from 1.85 to 5.55 kBq/µg for different labeled batches. The labeling efficiency (LE) was calculated as the percentage of [^225^Ac]Ac-DOTA-trastuzumab recovered divided by the amount of ^225^Ac used in the labeling reaction. Irrelevant DOTA-IgG_1_ was labeled with ^111^In at a SA of 0.111 MBq/µg or with ^225^Ac at 1.85–5.55 kBq/µg, respectively. The final RCP of ^225^Ac-labeled RICs was measured by ITLC-SG developed in 0.1 M sodium citrate buffer, pH 5.5. The R_f_ values of [^225^Ac]Ac-DOTA-trastuzumab and [^225^Ac]Ac-DOTA or free ^225^Ac were 0.0 and 1.0, respectively. The strips were divided into two sections (origin and solvent front) and measured in a γ-counter (Model 1480; PerkinElmer, Waltham, MA) using the γ-emissions (Eγ = 441 keV) of the ^213^Bi daughter product of ^225^Ac.

## Cell binding of radioimmunoconjugates (RICs)

The HER2 binding properties of [^111^In]In-DOTA-trastuzumab were assessed in a direct (saturation) radioligand cell-binding assay. The HER2-binding of [^225^Ac]Ac-DOTA-trastuzumab was not determined since it was assumed that substitution of ^225^Ac for ^111^In would not change these properties. Briefly, 1 × 10^6^ SK-BR-3 cells suspended in phosphate buffered saline (PBS), pH 7.4 in 1.5 mL Microtubes (Diamed Lab Supplies, Mississauga, ON, Canada) were incubated with increasing concentrations (0.073 to 300 nmoles/L) of [^111^In]In-DOTA-trastuzumab in PBS, pH 7.4 in the absence or presence of a 50-fold molar excess of trastuzumab for 3.5 h at 4 °C to measure total binding (TB) and non-specific binding (NSB), respectively. The tubes were mixed by gentle agitation every 30 min to minimize adsorpton of [^111^In]In-DOTA-trastuzumab onto the walls of the tubes. The tubes were centrifuged at 2000 rpm (400×*g*) for 5 min on an Eppendorf Centrifuge Model 5424 (Thermo Fisher Scientific), and the supernatant was collected. Cell pellets were then rinsed with PBS and centrifuged again to collect the supernatant. This procedure was repeated once more. Once the supernatant from all rinses and the cell pellet was isolated, the combined supernatant containing unbound activity was transferred using a micropipettte to a γ-counting tube. The cells were resuspended in 500 µL of ice-cold PBS, pH 7.4 and recentrifuged. The supernatant was removed and added to the previously recovered supernatant. This process was repeated a total of two times. The combined supernatants and cell pellets were measured in a γ-counter. NSB was subtracted from TB to obtain specific binding (SB). [^111^In]In-DOTA-trastuzumab specifically bound to SK-BR-3 cells (pmoles) was plotted versus the concentration of free (unbound) [^111^In]In-DOTA-trastuzumab (nmoles/L) and the resulting curve fitted to a one-site-receptor-binding model using Prism Ver. 8.0 software (GraphPad, San Diego, CA, USA) to estimate the dissociation constant (K_D_) and maximum number of binding sites per cell (B_max_).

## Cytotoxicity and DNA DSBs in vitro

The cytotoxicity of [^225^Ac]Ac-DOTA-trastuzumab on HER2-overexpressing BC cells in vitro was determined in a clonogenic survival assay. SK-BR-3 cells were selected for these assays due to their ability to form colonies that have strong adherence to 6- and 24-well plates during methylene blue staining making them suitable for clonogenic survival assays, while 164/8-1B/H2N.luc^+^ human BC cells which were used to estabish tumour xenografts in NRG mice did not adhere well to plates, and were not practical for conducting clonogenic survival assays. Briefly, 1.0 × 10^5^ SK-BR-3 cells were cultured overnight in wells in a 24-well plate (Sarstedt, Nümbrecht, Germany), then treated for 3 h with 20 nmoles/L (3.0 µg/mL) of [^225^Ac]Ac-DOTA-trastuzumab (SA = 0.148–1.665 kBq/µg) or DOTA-trastuzumab in 300 µL of medium. To assess HER2-specific cytotoxicity, cells were exposed to [^225^Ac]Ac-DOTA-trastuzumab (SA = 1.665 kBq/µg) in the presence of a 100-fold molar excess of DOTA-trastuzumab to block HER2. Then 1,800–6,000 cells were seeded into wells in 6-well culture plates (Sarstedt) containing 3 mL of fresh medium supplemented with 10% FBS per well to obtain a measurable number of colonies after culturing for 12 d at 37 °C and 5% CO_2_. Colonies were stained and fixed with methylene blue (1% in a 1:1 mixture of ethanol and water) and imaged on a ChemiDoc gel imaging system (Bio-Rad, Mississauga, ON, Canada). Surviving colonies (≥ 50 cells) were counted using ImageJ software (U.S. National Institutes of Health, Bethesda, MD, USA) and a customized macro (Cai et al. 2011). The plating efficiency (PE) was calculated by dividing the number of colonies by the number of cells seeded. The surviving fraction (SF) was calculated as the PE of treated cells divided by the PE of untreated cells. The SF was plotted versus SA of [^225^Ac]Ac-DOTA-trastuzumab or absorbed dose (D) in the cell (Gy) calculated as described under *Cellular Dosimetry*. The SF versus dose curve was fitted to a linear quadratic equation: $SF = {e}^{-\alpha D-\beta {D}^{2}}$, where α and β are curve fitting parameters.

DNA DSBs in SK-BR-3 cells caused by [^225^Ac]Ac-DOTA-trastuzumab were assessed by immunofluorescence confocal microscopy probing for phosphorylated histone-2AX (γ-H2AX) which accumulates at sites of unrepaired DSBs in the nucleus (Cai et al. 2009). Again, these assays were performed using SK-BR-3 cells instead of 164/8-1B/H2N.luc^+^ cells due to their greater adherence to glass coverslips. Briefly, 1 × 10^5^ SK-BR-3 cells were seeded onto glass coverslips (Ted Pella, Redding, CA, USA) placed in wells of 24-well plates (Sarstedt). After culturing overnight at 37 °C, the medium was replaced with 20 nmoles/L (3.0 µg/mL) of [^225^Ac]Ac-DOTA-trastuzumab (SA = 0.148–1.665 kBq/µg), DOTA-trastuzumab, or [^225^Ac]Ac-DOTA-trastuzumab (SA = 1.665 kBq/µg) mixed with a 100-fold molar excess of DOTA-trastuzumab to block HER2, in 300 μL of culture medium for 180 min. Cells were fixed, permeabilized, and blocked by BSA-donkey serum (Sigma-Aldrich) prior to being probed with anti-phospho-histone H2AX (Ser139, clone JBW301) mouse IgG_1_ (Upstate Biotechnology, Billerica, MA, USA) as previously reported (Cai et al. 2009). Cells were then incubated with Alexa Fluor 488 donkey anti-mouse IgG (H + L) (Invitrogen Molecular Probes, Carlsbad, CA). Vectashield® Mounting Medium containing 4',6-diamidino-2-phenylindole (DAPI) was used to mount cover slips onto microscope slides and stain cell nuclei. Images of γ-H2AX foci and nuclei were acquired with a confocal microscope (LSM 700) fitted with a Plan-Apochromat 63 × /1.4 Oil DIC M27 objective and laser scanning mode set as plane with two solid state laser lines (405 and 488 nm) chosen on the acquisition software LSM Zen 2011. At least 30 cells were imaged per slide, and an optical section (~ 1.2 μm) through the center of the nucleus was imaged. The laser intensity, signal amplification and offset, and image resolution (0.1 μm/pixel) were kept constant for all image acquisitions. Images were processed to calculate the mean integrated density of γ-H2AX foci per unit nucleus area using ImageJ.

## Cellular dosimetry

The radiation absorbed dose in the cell from treatment of SK-BR-3 cells in vitro in clonogenic survival and γ-H2AX assays with [^225^Ac]Ac-DOTA-trastuzumab was estimated based on the cellular uptake of ^225^Ac, which was derived by measurement of the cell uptake of [^111^In]In-DOTA-trastuzumab. Briefly, 1 × 10^5^ SK-BR-3 cells were seeded into wells in 24-well plates in 300 μL of medium and cultured overnight. The medium was then replaced with 300 μL of fresh medium containing 20 nmoles/L (3.0 µg/mL) of [^111^In]In-DOTA-trastuzumab at a SA of 37, 74 and 111 kBq/µg, and cells were incubated for 60, 120 or 180 min at 37 °C and 5% CO_2_. The culture medium containing unbound activity was removed. The wells were rinsed twice with cold PBS, pH 7.4. The cells were then lysed with 300 µL/well of 0.1 M NaOH at RT for 15 min and the cell lysates transferred to γ-counting tubes. The wells were rinsed twice with double-distilled water (ddH_2_O) and the rinses were added to the cell lysates. Cell bound activity in the lysates was measured in a γ-counter. Uptake of [^111^In]In-DOTA-trastuzumab (74 kBq/µg) by SK-BR-3 cells incubated with a 100-fold molar excess of trastuzumab was determined to assess HER2-specific cell uptake. Cells incubated with medium alone were detached at 60, 120 or 180 min by trypsinization and counted by a TC20 automated cell counter (Bio-Rad, Mississauga, ON, Canada) to estimate the number of cells per well for incubation with the RICs. The cellular uptake of [^111^In]In-DOTA-trastuzumab at 60, 120 or 180 min was expressed as percent incubated activity [%IA(t)]. Assuming that [^225^Ac]Ac-DOTA-trastuzumab exhibits the same uptake kinetics as [^111^In]In-DOTA-trastuzumab at the same mass concentration (20 nmoles/L; 3.0 µg/mL), ^225^Ac activity in SK-BR-3 cells (A_Cell_; Bq/cell) or in the surrounding medium (A_M_; Bq) at each incubation time point was calculated using Eq. 1 and 2, respectively:

1 $${\text{A}}_{{{\text{Cell}}}} ({\text{t}}) = {\text{IA}} \times {\text{IA}}\% ({\text{t}})/100 \times \exp ( - {\text{kt}})$$

2 $${\text{A}}_{{\text{M}}} ({\text{t}}) = {\text{IA}} \times \exp ( - {\text{kt}}){-}{\text{A}}_{{{\text{Cell}}}} ({\text{t}})$$

where IA was the ^225^Ac activity incubated with the cells (Bq), IA% is the percentage of activity taken up by cells at the selected incubation times, t (min), and k is the decay constant of ^225^Ac (4.81 × 10^–5^ min^−1^).

The time-integrated ^225^Ac activity in monolayer SK-BR-3 cells (Bq × min/cell) and in the surrounding medium during the 180-min incubation period (Ã_ML, Cell, 0-180 min_, Ã_M, 0-180 min_, respectively) were calculated from the area under curve (AUC) of a plot of A_Cell_ (t)/cell or A_M_ (t) versus time (min). The time-integrated activity of ^225^Ac in SK-BR-3 cells for the 12-d colony formation period (Ã_Cell180min-12d_; Bq × min/cell) was calculated using Eqs. 3–5:

3 $${\text{T}}_{{\text{e}}} = {\text{T}}_{{\text{b}}} {\text{T}}_{{\text{p}}} /\left( {{\text{T}}_{{\text{b}}} + {\text{T}}_{{\text{p}}} } \right)$$

4 $${\text{k}}_{{\text{e}}} = \ln \left( 2 \right)/{\text{Te}}$$

5 $$\ A_{{{\text{Cell}},180\,\min - 12{\text{d}}}} = {\text{A}}_{{{\text{Cell}}}} \left( {180\,\min } \right)/{\text{k}}_{{\text{e}}} \times [1 - \exp ( - 12 \times 24 \times 60\,{\text{k}}_{{\text{e}}} )]$$

where, T_b_ the biological half life, equivalent to the doubling time of SK-BR-3 cells (1.55 d = 2246 min) and T_p_ is the physical half life of ^225^Ac (10 d = 14,400 min). Thus, the effective half life (T_e_) and decay constant (k_e_) were calculated as 1,943 min and 3.568 × 10^–4^ min^−1^. Ã in Bq × min was converted to Bq × s by multiplying by the unit conversion factor = 60 s/min.

During the colony formation period, since the cells were seeded in fresh medium, Ã_M, 3 h-12d_ was assumed as zero.

The radiation absorbed dose in SK-BR-3 cells (modeled as 18 µm diameter spheres closely packed as a monolayer) in wells in a 24-well plate incubated with [^225^Ac]Ac -DOTA-trastuzumab (20 nmoles/L, 3.7 µg/mL, 0–111 kBq/µg) in 300 µL of medium (modeled as cylinder of 1.54 cm diameter and 0.161 cm high) for 180 min, followed by 12 d colony formation from a single seeded cell (modeled as 18 µm diameter sphere) was estimated by cellular dosimetry. S values of cell to cell (S_C←C_), cell monolayer to cell (S_C←CML_) and medium to cell (S_C←M_) were calculated using Monte Carlo N-Particle (MCNP) code Ver. 6.1 (Los Alamos National Laboratory, Los Alamos, NM, USA) (Cai et al. 2017). It was assumed that ^225^Ac was homogenously distributed in the cell, the cell monolayer or cylinder, and all ^225^Ac decay daughters remained at the same location as ^225^Ac. The α-particle emission spectra for ^225^Ac and its daughters were obtained from the Medical Internal Radiation Dose (MIRD) decay schemes (Eckerman and Endo 2008) and used as particle input in the simulation. 5 × 10^6^ α-particles were launched for each simulation and the energies deposited in a single cell, monolayer cells and medium were tallied to calculate values of S_C←C_, S_C←CML_ and S_C←M_ in Gy × Bq^−1^ s^−1^. Neither photon nor electron emissions were included in the calculation for simplicity, since the energy of α-particle emission per nuclear transition of ^225^Ac including its daughters (27.996 MeV) is 900 times greater than the energy of all emitted electrons and photons (0.0311 MeV) (Eckerman and Endo 2008). The doses (D_SF_ in Gy) in the cell (target) from three sources (medium, cell monolayer, cell) during the clonogenic assay were calculated using Eq. (6):

6 $${\text{D}} = \ A_{{{\text{M}},0 - 180\min }} \times {\text{S}}_{{{\text{C}} \leftarrow {\text{M}}}} + \ A_{{{\text{CML}},0 - 180\min }} \times {\text{S}}_{{{\text{C}} \leftarrow {\text{CML}}}} + \ A_{{{\text{C}},180\min - 12{\text{d}}}} \times {\text{S}}_{{{\text{C}} \leftarrow {\text{C}}}}$$

where the units of Ã were Bq × s.

The doses (D γ-_H2AX_ in Gy) in the cell (target) from two sources (medium, cell monolayer) during the γ-H2AX assay were calculated using Eq. (7):

7 $${\text{D}}_{{{\upgamma{}} - {\text{H}}2{\text{AX}}}} = \ A_{{{\text{M}},0 - 180\min }} \times {\text{S}}_{{{\text{C}} \leftarrow {\text{M}}}} + \ A_{{{\text{CML}},0 - 180\min }} \times {\text{S}}_{{{\text{C}} \leftarrow {\text{CML}}}}$$

## Biodistribution and SPECT/CT imaging studies

Biodistribution studies were performed in NRG mice with s.c. HER2-overexpressing 164/8-1B/H2N.luc + human BC xenografts coinjected intravenously (i.v.; tail vein) with 7–8 MBq (20 µg) of [^111^In]In-DOTA-trastuzumab mixed with DOTA-trastuzumab (18.5 µg) and 4 kBq (1.5 µg) of [^225^Ac]Ac-DOTA-trastuzumab (total mass = 40 µg). [^111^In]In-DOTA-trastuzumab was used to calculate the uptake of [^225^Ac]Ac-DOTA-trastuzumab in tissues by γ-counting due to the very low amounts of ^225^Ac (4 kBq) that may be safely administered to mice and due to inaccuracies that could be introduced by deriving ^225^Ac concentrations using the γ-emissions (Eγ = 441 keV) of the ^213^Bi daughter of ^225^Ac. Thus, these studies assumed that the biodistribution of [^111^In]In-DOTA-trastuzumab traced that of [^225^Ac]Ac-DOTA-trastuzumab when administered simultaneously in a combined total mass amount (40 µg). Co-injection of [^111^In]In-DOTA-trastuzumab further enabled single photon emission computed tomography/computed tomography (SPECT/CT) imaging to assess uptake of the RICs into 164/8-1B/H2N.luc + human BC xenografts and normal organs. Groups of 5 NRG mice with s.c. 164/8-1B/H2N.luc^+^ tumour xenografts were coinjected with [^111^In]In-DOTA-trastuzumab and [^225^Ac]Ac-DOTA-trastuzumab as indicated above or were co-injected with 7–8 MBq (38.5 µg) of irrelevant [^111^In]In-DOTA-IgG_1_ and 4 kBq (1.5 µg) of [^225^Ac]Ac-DOTA-IgG_1_ (total mass = 40 µg) in 160 µL of 0.9% Sodium Choride Injection, USP. At 48 h postinjection (p.i.) 3 mice per group were imaged by SPECT/CT, then all mice were sacrificed under 2% isoflurane in O_2_ anaesthesia and the tumour and samples of blood and other normal tissues obtained, weighed and ^111^In measured in a γ-counter. Tissue uptake of ^111^In was expressed as percent injected dose/g (%ID/g). Mice were anaesthetized using 2% isoflurane in O_2_ and were imaged in a prone position on a trimodality NanoScan® SPECT/CT/PET system (Mediso, Budapest, Hungary) equipped with 4 NaI (Tl) detectors. SPECT employed a 40 s/frame acquisition time resulting in a scan duration of 45 min. CT images were acquired using parameters of 50 kVp X-rays, 980 μA and 300 ms exposure time, isotropic voxel size of 125 μm and maximum field-of-view with 1:4 binning. Images were reconstructed using TeraTomo 3D Normal Dynamic Range Monte Carlo-based reconstruction protocol with a 128 × 128 reconstruction matrix, with three subsets of data undergoing 48 iterations applied with CT-based attenuation and scatter correction. SPECT and CT images were co-registered by InterView Fusion software (Ver. 3.09; Mediso).

## Statistical analysis

Data were expressed as mean ± SD. Statistical analyses were performed using Prism Ver. 9.5 software (GraphPad) with significance tested by Welch’s t-test or one-way ANOVA (*P* < 0.05).

## Results

### Radioimmunoconjugates (RICs)

**Fig. 1:**
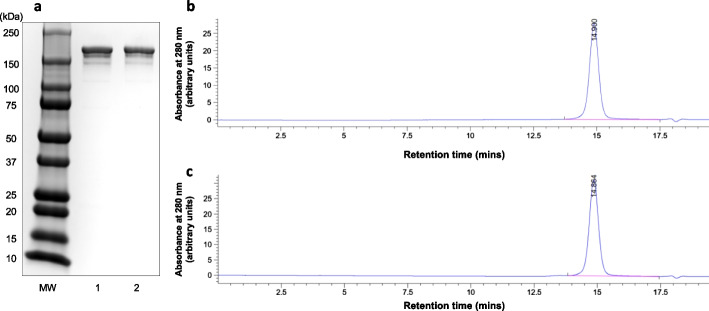
**a** SDS-PAGE of trastuzumab (lane 1) and DOTA-trastuzumab (lane 2) under non-reducing conditions on a 4–20% Mini-Protean Tris/glycine mini-gel. Bands were stained with Coomassie Brilliant Blue G-250. MW: Broad range MW markers. SE-HPLC analysis of **b** trastuzumab or **c** DOTA-trastuzumab on a BioSep SEC-S2000 column eluted with 0.1 M NaH_2_PO_4_ buffer, pH 7.0 at a flow rate of 0.8 mL/min with UV detection at 280 nm

**Fig. 2:**
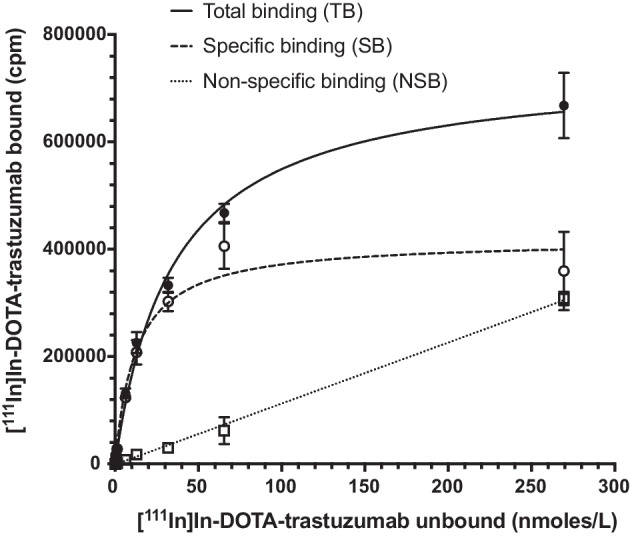
Binding of [^111^In]In-DOTA-trastuzumab to HER2-positive SK-BR-3 human BC cells in the absence (TB) or presence (NSB) of a 50-fold molar excess of trastuzumab. The SB was calculated by subtracting NSB from TB. Fitting the SB curve to a 1-site receptor binding model estimated the K_D_ = 1.2 ± 0.3 × 10^–8^ mol/L and B_max_ = 4.2 ± 0.2 × 10^6^ receptors/cell

Reaction of trastuzumab (30 mg/mL) with DOTA-NHS at a 30:1 molar excess resulted in conjugation of 12.7 ± 1.2 DOTA per trastuzumab molecule measured by MALDI-TOF analysis. Reaction of trastuzumab at a 10:1, 30:1 and 60:1 molar excess resulted in 1.9 ± 0.7. 5.5 ± 2.2 and 10.1 ± 1.9 DOTA per trastuzumab molecule measured by ITLC-SG after trace labeling with ^111^In (Additional file 1: Fig. S1). DOTA-trastuzumab and trastuzumab migrated as a single major protein band by SDS-PAGE (MW ~ 185 kDa) (Fig. 1a). SE-HPLC with UV detection at 280 nm showed a single peak for trastuzumab (Fig. 1b) and DOTA-trastuzumab (Fig. 1c) with similar retention times (t_R_) of 14.90 and 14.86 min, respectively. Immunoconjugates synthesized by reaction of trastuzumab (15 mg/mL) with a 60-fold molar excess of DOTA-NHS were used to study the effects of incubation temperature and time, SA and protein concentration on LE with ^111^In. There was a trend towards higher LE with ^111^In at increasing temperature with the highest LE (95.2 ± 1.2%) obtained at 1.5 h and 40 °C and SA = 0.1 MBq/µg (Additional file 1: Fig. S2a). There were no significant differences (*P* > 0.05) in ^111^In LE for 0.5, 1, 1.5, 2 or 3 h incubation times at 40 °C and SA = 0.1 MBq/µg (Additional file 1: Fig. S2b). There were no significant differences in LE with ^111^In over a SA range of 0.1 MBq/µg to 0.25 MBq/µg at 40 °C for 0.5 h (Additional file 1: Fig. S2c). Protein concentration did not affect LE with ^111^In over a concentration range of 2.5–15.0 mg/mL at 40 °C for 0.5 h (Additional file 1: Fig. S2d). Based on these results, DOTA-trastuzumab synthesized by reaction of trastuzumab (30 mg/mL) with a 30:1 molar excess of DOTA-NHS resulting in 12.7 ± 1.2 DOTA conjugated per trastuzumab molecule and was labeled with ^111^In by incubation for 0.5 h at 40 °C at a SA = 0.1–0.35 MBq/µg and protein concentration = 15 mg/mL for all subsequent studies. The LE and final RCP of [^111^In]In-DOTA-trastuzumab labeled under these conditions was 94.3 ± 8.1%. The LE of DOTA-trastuzumab prepared at 10:1, 30:1 or 60:1 molar excess of DOTA-NHS:trastuzumab under these same conditions except for a longer 1.5 h incubation time was 90.7 ± 0.1%, 93.8 ± 1.6% and 95.2 ± 1.2%, respectively (Additional file 1: Fig. S3). The conditions chosen for ^225^Ac labeling of DOTA-trastuzumab (37 °C for 2 h at a protein concentration of 5 mg/mL) were based on the protocol described by Macguire et al. (Maguire et al. 2014). Under these conditions, the LE of DOTA-trastuzumab with ^225^Ac at a SA = 5.3 kBq/µg was 76.4 ± 3.1%; (n = 3). Following purification by ultrafiltration on an Amicon Ultra 0.5 device, the final RCP of [^225^Ac]Ac-DOTA-trastuzumab was 95.9 ± 0.9 (n = 3). Irrelevant human IgG_1_ was labeled with ^111^In or ^225^Ac to a final RCP of 95.7 ± 0.1% and 94.4 ± 0.2%, respectively. [^111^In]In-DOTA-trastuzumab exhibited saturable binding to HER2-positive SK-BR-3 cells that was blocked by a 50-fold molar excess of trastuzumab (Fig. 2). Fitting of the SB curve to a 1-site receptor-binding model estimated the K_D_ = 1.2 ± 0.3 × 10^–8^ mol/L and B_max_ = 4.2 ± 0.2 × 10^5^ receptors/cell.

## Cytotoxicity and DNA DSBs

**Fig. 3:**
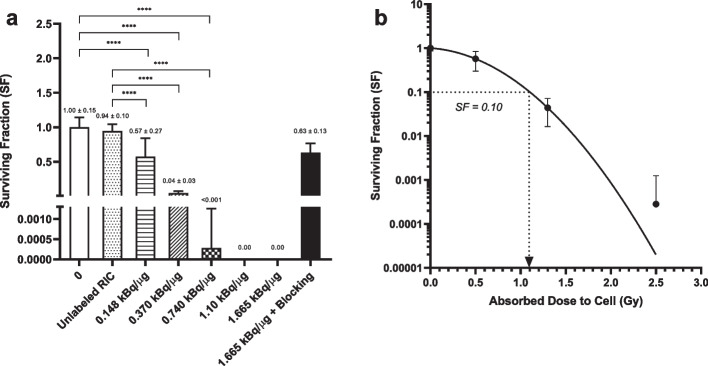
**A** Surviving fraction (SF) of HER2-positive human BC cells exposed in vitro to medium, DOTA-trasuzumab, [^225^Ac]Ac-DOTA-trastuzumab labeled at increasing SA (0.148–1.665 kBq/µg) or [^225^Ac]Ac-DOTA-trastuzumab (SA = 1.665 kBq/µg) combined with a 100-fold excess of DOTA-trastuzumab to block HER2. Values shown are the mean ± SD (n = 6–12). Significant differences (****P ≤ 0.0001) are indicated by the asterisks. **B** SF versus dose in SK-BR-3 cells estimated by cellular dosimetry. The curve was fitted to a linear quadratic model. The estimated dose to decrease the SF to 0.10 (dotted line) was 1.10 Gy

The SF of SK-BR-3 cells treated in vitro with [^225^Ac]Ac-DOTA-trastuzumab at increasing SA (0.148–1.665 kBq/µg) or [^225^Ac]Ac-DOTA-trastuzumab (SA = 1.665 kBq/µg) mixed with a 100-fold molar excess of DOTA-trastuzumab to block HER2 is shown in Fig. 3a. [^225^Ac]Ac-DOTA-trastuzumab was potently cytotoxic, significantly decreasing the SF of SK-BR-3 cells compared to medium treated cells to 0.57 ± 0.10 at a SA = 0.148 kBq/µg (*P* < 0.0001), 0.04 ± 0.03 at a SA = 0.37 kBq/µg (*P* < 0.0001) and < 0.001 at a SA = 0.74 kBq/µg (*P* < 0.0001). No surviving colonies were found at SA = 1.10 kBq/µg or 1.665 kBq/µg. Blocking HER2 with an excess of DOTA-trastuzumab restored the SF of SK-BR-3 cells treated with [^225^Ac]Ac-DOTA-trastuzumab (SA = 1.665 kBq/µg) to 0.63 ± 0.13, demonstrating that the cytotoxicity of [^225^Ac]Ac-DOTA-trastuzumab was HER2-specific. Treatment of SK-BR-3 cells with DOTA-trastuzumab had no significant effect on SF compared to cells treated with medium (SF = 0.94 ± 0.10 vs. 1.00 ± 0.15; *P* > 0.05). Based on the estimated absorbed dose in SK-BR-3 cells treated with [^225^Ac]Ac-DOTA-trastuzumab (see *Cellular Dosimetry* results), the SF was plotted versus dose (Gy) in Fig. 3b. The survival curve was fitted to a linear quadratic model, $SF = {e}^{-\alpha D-\beta {D}^{2}}$, where D is the dose in the cell and α = 0.3214 Gy^−2^ and β = 1.604 Gy^−2^ are fitting parameters. The estimated dose required to decrease the SF to 0.10 (D_10_), based on the curve fit was 1.10 Gy.

**Fig. 4:**
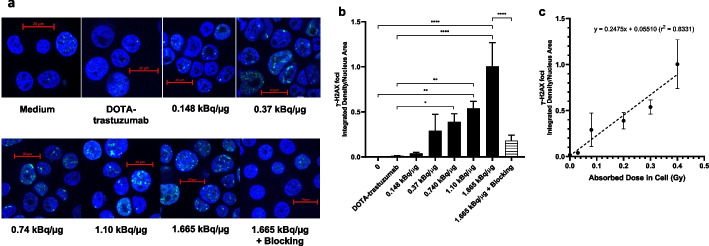
**a** DNA DSBs visualized by immunofluorescence microscopy probing for γ-H2AX foci in the nucleus of SK-BR-3 cells treated in vitro with medium, DOTA-trastuzumab or [^225^Ac]Ac-DOTA-trastuzumab at increasing specific activity (SA = 0.148–1.665 kBq/µg) or [^225^Ac]Ac-DOTA-trastuzumab (SA-1.665 kBq/µg) combined with a 100-fold excess of DOTA-trastuzumab to block HER2. **b** Integrated density of γ-H2AX foci in the nucleus of SK-BR-3 cells receiving these treatments. Significant differences are noted by the asterisks (*P ≤ 0.05, **P ≤ 0.01, ****P ≤ 0.0001). **c.** Correlation between the integrated density of γ-H2AX foci in the nucleus of SK-BR-3 cells versus absorbed dose deposited in the cells by [^225^Ac]Ac-DOTA-trastuzumab

[^225^Ac]Ac-DOTA-trastuzumab caused DNA DSBs in SK-BR-3 cells detected by immunofluorescence microscopy for γ-H2AX (Fig. 4a). Quantification of the integrated density of γ-H2AX foci in the nucleus (Fig. 4b) showed that DNA DSBs increased with increasing SA (0.148–1.665 kBq/µg) of [^225^Ac]Ac-DOTA-trastuzumab. The density of γ-H2AX foci in SK-BR-3 cells exposed to [^225^Ac]Ac-DOTA-trastuzumab at a SA = 0.74 kBq/µg or 1.665 kBq/µg was 22-fold (*P* = 0.016) and 57-fold (*P* = 0.023) significantly higher, respectively than in cells treated with medium. The density of γ-H2AX foci in cells exposed to [^225^Ac]Ac-DOTA-trastuzumab at SA = 0.74, 1.10 or 1.665 kBq/µg was 36-fold (*P* = 0.017), 50-fold (*P* = 0.007) and 94-fold (*P* = 0.023) significantly greater, respectively than cells treated with DOTA-trastuzumab. Blocking HER2 with an excess of DOTA-trastuzumab significantly decreased the density of γ-H2AX foci in the nucleus by 5.5-fold (*P* = 0.023) compared to cells exposed to [^225^Ac]Ac-DOTA-trastuzumab at a SA = 1.665 kBq/µg. The density of γ-H2AX foci in the nucleus was directly correlated with the absorbed dose in SK-BR-3 cells treated with [^225^Ac]Ac-DOTA-trastuzumab (see *Cellular Dosimetry*; Fig. 4c).

## Cellular dosimetry

The absorbed dose in SK-BR-3 cells exposed to ^[225^Ac]Ac-DOTA-trastuzumab (SA = 0.148–1.665 kBq/µg) in clonogenic survival assays was estimated based on the cellular uptake of [^111^In]In-DOTA-trastuzumab since the activity of ^225^Ac incubated with the cells was too low to measure accurately. [^111^In]In-DOTA-trastuzumab (SA = 0.037–0.111 MBq/µg) was bound by SK-BR-3 cells, reaching a maximum at 120 min after incubation, but was slightly lower at 180 min (Additional file 1: Fig. S4). Since [^225^Ac]Ac-DOTA-trastuzumab was labeled at a different SA than [^111^In]In-DOTA-trastuzumab, the effect of SA on uptake by SK-BR-3 cells was examined. There was no effect of SA on cellular uptake (Additional file 1: Fig. S4). Incubation of SK-BR-3 cells with [^111^In]In-DOTA-trastuzumab mixed with a 100-fold molar excess of DOTA-trastuzumab to block HER2 greatly decreased uptake by SK-BR-3 cells (Fig. S4), demonstrating that uptake was HER2-specific. The time-integrated activity of ^225^Ac in the medium (Ã_M,0-180min_) and cell monolayer (Ã_CML,0-180min_) source compartments in the 180 min treatment period and in the cells (Ã_C,180min-12d_) during the 12 d colony-forming period, derived from ^111^In measurements at each SA is shown in Supporting Information, Table S1. The average values of time-integrated activities of ^225^Ac per cell over SA = 0.037–0.111 MBq/µg were used in the calculation of absorbed dose to SK-BR-3 cells. The S-values for each compartment, S_C←M_, S_C←CML_, and S_C←C_ calculated using MCNP modeling were 4.37 × 10^–9^, 0.1114 and 0.1086 GyBq^−1^s^−1^, respectively. Assuming treatment with 1 Bq (20 nmoles/L; 3.0 µg/mL) of [^225^Ac]Ac-DOTA-trastuzumab contained in 300 µL of medium in a well in a 24-well plate, the absorbed doses in SK-BR-3 cells during the treatment and colony-forming periods and at different SA are shown in Table 1. The total absorbed dose (Gy) in SK-BR-cells during the clonogenic assay was 0.5, 1.3, 2.5, 4, and 6 Gy for treatment with [^225^Ac]Ac-DOTA-trastuzumab at SA = 0.15, 0.37, 0.74, 1.10 and 1.66 kBq/µg, respectively. The total absorbed dose (Gy) in SK-BR-cells during γ-H2AX assay was 0.03, 0.08, 0.2, 0.3, and 0.4 Gy for treatment with [^225^Ac]Ac-DOTA-trastuzumab at a SA = 0.148, 0.37, 0.74, 1.10 and 1.665 kBq/µg, respectively.

**Table 1:** Absorbed doses in HER2-positive SK-BR-3 cells exposed in vitro to [^225^Ac]Ac-DOTA-trastuzumab labeled at increasing specific activity (SA) in clonogenic survival assays ^a^

SA (kBq/µg)	^225^Ac per well (Bq) ^b^	Absorbed dose from 180 min treatment period (Gy)		Absorbed dose from 12 d colony-forming period (Gy)	Total absorbed dose (Gy)
		From monolayer cells (Gy)	From medium (Gy)		
0.15	131	0.027 ± 0.002	0.006 ± 0.000	0.47 ± 0.13	0.50 ± 0.10
0.37	328	0.069 ± 0.004	0.015 ± 0.000	1.17 ± 0.33	1.25 ± 0.30
0.74	655	0.137 ± 0.009	0.030 ± 0.000	2.35 ± 0.66	2.52 ± 0.70
1.10	982	0.206 ± 0.020	0.044 ± 0.000	3.52 ± 0.99	3.77 ± 1.00
1.66	1474	0.309 ± 0.020	0.067 ± 0.000	5.28 ± 1.49	5.66 ± 2.00

^a^Absorbed doses were estimated using the MIRD schema as described in the Methods section (Cellular Dosimetry)

^b^Assumes treatment of 1 × 10^5^ cells with 300 µL of 20 nmoles/L of [^225^Ac]Ac-DOTA-trastuzumab labeled at the indicated SA

## Biodistribution and SPECT/CT imaging studies

**Fig. 5:**
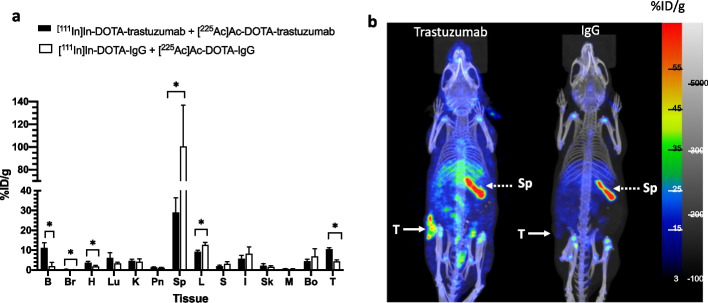
**a** Tumour and normal tissue uptake at 48 h p.i. of [^111^In]In-DOTA-trastuzumab (7–8 MBq) coinjected with [^225^Ac]Ac-DOTA-trastuzumab (4 kBq; total mass dose = 40 µg) in NRG mice with s.c. HER2-positive 164/8-1B/H2N.luc + human BC tumours. B: blood, Br: brain, H: heart, Lu: lungs, K: kidneys, Pn: pancreas, Sp: spleen, L: liver, S: stomach, I: intestine, Sk: skin, M: muscle, Bo: bone. Tissue activity was quantified by γ-counting of ^111^In. Values shown are the mean ± SD (n = 4–5). Significant differences (*P < 0.05) are indicated by asterisks. **b** Representative SPECT/CT images of tumour-bearing NRG mice at 48 h p.i. of [^111^In]In-DOTA-trastuzumab coinjected with [^225^Ac]Ac-DOTA-trastuzumab (left image) or [^111^In]In-DOTA-IgG_1_ coinjected with [^225^Ac]Ac-DOTA-IgG_1_ (right image). T: tumour, Sp: spleen. Scale for SPECT/CT images shows the percent injected dose/g (%ID/g). Also shown in grayscale is the scale for the CT image

The biodistribution of [^111^In]In-DOTA-trastuzumab (7–8 MBq; 20 µg) mixed with DOTA-trastuzumab (18 µg) and coinjected with 4 kBq (1.5 µg) of [^225^Ac]Ac-DOTA-trastuzumab (total mass = 40 µg) or equivalent amounts of irrelevant [^111^In]In-DOTA-IgG_1_ coinjected with [^225^Ac]Ac-DOTA-IgG_1_ (total mass = 40 µg) at 48 h p.i. in NRG mice with s.c. HER2-positive 164/8-1B/H2N.luc^+^ human BC tumours are shown in Fig. 5a. ^111^In was used to measure the tumour and normal tissue uptake of the RICs due to the very low amount (4 kBq) of ^225^Ac that could be safely injected, assuming that coadministered ^111^In and ^225^Ac-labeled RICs distribute equivalently. The γ-photons of ^111^In [Eγ = 171 keV (90.7%) and 245 keV (94.1%) were used to acquire SPECT/CT images (Fig. 5b). The tumour uptake of [^111^In]In-DOTA-trastuzumab was 2.5-fold significantly greater than irrelevant [^111^In]In-DOTA-IgG_1_ (10.6 ± 0.6% ID/g vs. 4.3 ± 0.7% ID/g respectively, *P* < 0.0001). The highest normal tissue uptake was in the spleen but this was 3.5-fold significantly lower for [^111^In]In-DOTA-trastuzumab than [^111^In]In-DOTA-IgG_1_ (28.9 ± 7.4 vs. 100.6 ± 36.2% ID/g; *P* = 0.003). Liver uptake of [^111^In]In-DOTA-trastuzumab was 1.4-fold significantly lower than [^111^In]In-DOTA-IgG_1_ (9.2 ± 0.8 vs. 12.6 ± 1.2% ID/g; *P* = 0.001). Blood and heart activity were 4.1-fold and 2.0-fold significantly higher for [^111^In]In-DOTA-trastuzumab than [^111^In]In-DOTA-IgG_1_, respectively (11.1 ± 2.7 vs. 1.9 ± 1.9% ID/g; *P* < 0.001 and 3.7 ± 0.7 vs. 1.9 ± 0.5; *P* = 0.002, respectively). Tumours were well-visualized by SPECT/CT in mice at 48 h post co-injection of [^111^In]In-DOTA-trastuzumab and [^225^Ac]Ac-DOTA-trastuzumab, but not in mice co-injected with [^111^In]In-DOTA-IgG_1_ and [^225^Ac]Ac-DOTA-IgG_1_ (Fig. 5b).

## Discussion

[^225^Ac]Ac-DOTA-trastuzumab was synthesized and characterized, and the effect of treatment of HER2-positive SK-BR-3 human BC cells in vitro with [^225^Ac]Ac-DOTA-trastuzumab on their clonogenic survival or for causing DNA DSBs assessed. The radiation absorbed dose in SK-BR-3 cells treated with [^225^Ac]Ac-DOTA-trastuzumab was estimated and the relationship between dose and SF and DNA DSBs determined. Reaction of trastuzumab (15 mg/mL) with a 30:1 molar excess of DOTA-NHS, resulted in conjugation of 12.7 ± 1.2 DOTA per trastuzumab molecule measured by MALDI-TOF analysis. Lower DOTA conjugation levels (5.5 ± 1.3 DOTA/trastuzumab were measured by ITLC-SG after trace labeling of the conjugation sample with ^111^In (Additional file 1: Fig. S1), but MALDI-TOF is considered to be more accurate, since it directly measures the increase in molecular weight associated with DOTA conjugation of trastuzumab. Despite this relatively high level of DOTA modification, the immunoconjugates migrated as a single band on SDS-PAGE and a single peak by SE-HPLC (Fig. 1). In addition following labeling with ^111^In, high affinity specific binding of [^111^In]In-DOTA-trastuzumab to HER2-positive SK-BR-3 cells was observed (Fig. 2). The K_D_ of [^111^In]In-DOTA-trastuzumab (1.2 ± 0.3 × 10^–8^ mol/L) was approximately two-fold greater than previously reported for trastuzumab (5 × 10^–9^ mol/L). DOTA-NHS reacts with the ε-amino group on lysine amino acids or the N-terminal amines of trastuzumab. There are four N-terminal amines – one on each of the two heavy and light chains and 88 lysines in the trastuzumab molecule, but only one lysine is present in the complementarity-determining region (CDR) at heavy chain position K^65^ (Chen et al. 2016). This may explain the minimal impact of trastuzumab modification with multiple DOTA chelators on HER2-binding affinity. Guleria M et al. measured a similar K_D_ (1.36 × 10^–8^ mol/L) for binding of [^177^Lu]Lu-DOTA-trastuzumab conjugated with 6 DOTA to HER2-positive SK-OV-3 human ovarian cancer cells (Guleria et al. 2021). DOTA is often used to complex ^225^Ac to biomolecules, since DOTA forms stable complexes with tripositive metal ions (ie. Ac^3+^) (Viola-Villegas and Doyle, 2009). However, due to the lower stability of DOTA complexes with metals that have a large ionic radius (e.g. Ac^3+^), novel chelators for ^225^Ac have recently been reported (e.g. macropa, H_2_bispa2, H_4_octapa) (Hu and Wilson 2022; Ramogida et al. 2019). It would be interesting to compare these chelators to DOTA for ^225^Ac-labeling of trastuzumab in the future. The protocol described by Maguire et al. was followed for ^225^Ac labeling of DOTA-trastuzumab (Maguire et al. 2014). The LE of DOTA-trastuzumab (5 mg/mL) with ^225^Ac at a SA of SA = 5.3 kBq/µg after an incubation time of 2 h at 37 °C was 76.4 ± 3.1%. The LE of DOTA-trastuzumab with ^111^In at SA = 0.1 MBq/µg (89.8 ± 4.8%; Additional file 1: Fig. S1D) was higher under similar conditions (5 mg/mL, 0.5 h), but purification of [^225^Ac]Ac-DOTA-trastuzumab by ultrafiltration increased the final RCP of to 95.9 ± 0.9%.

Treatment of SK-BR-3 cells with [^225^Ac]Ac-DOTA-trastuzumab (20 nmoles/L; 3.7 µg/mL) potently decreased their SF in vitro dependent on SA (0.148–1.665 kBq/µg; Fig. 3a). At a SA = 0.754 kBq/µg, the SF was < 0.001 and SA = 1.10 or 1.665 kBq/µg, there were no surviving colonies detected. The cytotoxicity of [^225^Ac]Ac-DOTA-trastuzumab was HER2-specific, as SF was restored to 0.63 ± 0.13 at a SA = 1.665 kBq/µg by co-incubation of SK-BR-3 cells with a 100-fold excess of DOTA-trastuzumab to block HER2. Yoshida et al. reported that the SF of HER2-positive SUM225-Luc + ductal carcinoma in situ of the breast (DCIS) cells was decreased to 0.15 by exposure to 3.7 kBq/mL of [^225^Ac]Ac-DOTA-trastuzumab (SA = 37 kBq/µg) compared to 0.91 for exposure to 3.7 kBq/mL of irrelevant [^225^Ac]Ac-DOTA-rituximab, demonstrating HER2 specific cell killing (Yoshida et al. 2016). In our study, co-incubation of [^225^Ac]Ac-DOTA-trastuzumab with a 100-fold excess of trastuzumab to determine HER2-specific killing of SK-BR-3 cells may further represent treatment of these cells with trastuzumab. This may explain the residual cytotoxicity observed even in the presence of HER2 blocking (ie. SF = 0.63 ± 0.13). Yoshida reported that trastuzumab treatment (1 µg/mL) of SUM225-Luc+ cells decreased the SF to 0.65–0.7, but [^225^Ac]Ac-DOTA-trastuzumab was much more potent (SF = 0.1) (Yoshida et al. 2016). At a similar concentration as used in our study (4 µg/mL), Rasaneh et al. found using the MTT cell viability assay that treatment of SK-BR-3 cells in vitro with [^177^Lu]Lu-DOTA-trastuzumab (SA = 0.0925 MBq/µg) decreased the number of viable cells to 10%, while trastuzumab decreased the cell number to 41% (Rasaneh et al. 2010). Since the SF of SK-BR-3 cells exposed to [^225^Ac]Ac-DOTA-trastuzumab was decreased > 100-fold compared to treatment in vitro with [^177^Lu]Lu-DOTA-trastuzumab reported by Rasaneh et al. (Rasaneh et al. 2010) (ie. SF < 0.001 vs. 0.10, respectively) and this decreased SF was achieved at a 123-fold lower SA of [^225^Ac]Ac-DOTA-trastuzumab than [^177^Lu]Lu-DOTA-trastuzumab (ie. 0.754 kBq/µg vs. 0.0925 MBq/µg, respectively), these results indicate that the α-particles emitted by ^225^Ac were far more effective than the β-particles emitted by ^177^Lu for killing HER2-positive BC cells.

The cytotoxicity of [^225^Ac]Ac-DOTA-trastuzumab was further assessed by plotting the SF versus dose in SK-BR-3 cells (Fig. 3b). The dose was estimated from the time-integrated activity in SK-BR-3 cells derived from the cell uptake of [^111^In]In-DOTA-trastuzumab (Additional file 1: Fig. S4 and Table S1). The dose required to decrease the clonogenic survival of SK-BR-3 cells to 0.10 (D_10_) was 1.10 Gy for [^225^Ac]Ac-DOTA-trastuzumab versus D_10_ = 4.855 Gy reported for exposure of SK-BR-3 cells to γ-radiation (Cai et al. 2011). Relative biological effectiveness (RBE) is defined as the ratio of the dose of the reference radiation versus the test radiation required to produce an equivalent biologic effect (i.e. SF = 0.10). Based on a comparison with γ-radiation, the calculated RBE of [^225^Ac]Ac-DOTA-trastuzumab was 4.4 (Chen 2004). No previous studies have reported the RBE for [^225^Ac]Ac-DOTA-trastuzumab for killing HER2-positive BC cells. However, Nayak et al. estimated a RBE = 3.4 for Capan-2 human pancreatic cancer cells treated in vitro with α-particle emitting, [^213^Bi]Bi-DOTATOC versus γ-radiation, while the RBE of [^177^Lu]Lu-DOTATOC was 1.0 (Nayak et al. 2007).

[^225^Ac]Ac-DOTA-trastuzumab caused multiple DNA DSBs in SK-BR-3 cells assessed by immunofluorescence confocal microscopy probing for γ-H2AX (Cai et al. 2009) (Fig. 4a). The integrated density of γ-H2AX foci per cell nucleus area was directly dependent on the SA (0.148–1.665 kBq/µg) of [^225^Ac]Ac-DOTA-trastuzumab; Fig. 4b) and the absorbed dose in the cells (0–0.5 Gy, Fig. 4c). DNA DSBs were HER2-specific since coincubation of SK-BR-3 cells with [^225^Ac]Ac-DOTA-trastuzumab combined with a 100-fold excess of trastuzumab to block HER2 significantly decreased the integrated γ-H2AX foci density per nucleus area by 5.5-fold compared to [^225^Ac]Ac-DOTA-trastuzumab alone (SA = 1.665 kBq/µg). DNA DSBs in HER2-positive BC cells exposed to [^225^Ac]Ac-DOTA-trastuzumab have not been previously studied. However, Rodak et al. (2022) reported that treatment of SKOV-3 human ovarian cancer cells in vitro with 15–625 kBq/mL of [^225^Ac]Ac-DOTA-sdAb single domain antibody similarly caused multiple DNA DSBs.

Biodistribution and SPECT/CT imaging studies were performed in NRG mice with s.c. 164/8-1B/H2N.luc^+^ HER2-positive BC xenografts co-injected with 7–8 MBq of [^111^In]In-DOTA-trastuzumab and 4 kBq of [^225^Ac]Ac-DOTA-trastuzumab (total mass = 40 µg) since it was not feasible to measure these very small amounts of ^225^Ac by using the γ-emissions of the ^213^Bi daughter product. This assumes that [^111^In]In-DOTA-trastuzumab radiotraces the biodistribution of [^225^Ac]Ac-DOTA-trastuzumab. Borchardt et al. (2003) reported similar biodistribution of i.p. injected [^111^In]In-DOTA-trastuzumab and [^225^Ac]Ac-DOTA-trastuzuab in athymic nude mice with i.p. SK-OV-3 human ovarian cancer xenografts. However, due to possible differences in the stability of [^111^In]In-DOTA and [^225^Ac]Ac-DOTA complexes or due to the release and redistribution of daughter radionuclides following decay of ^225^Ac, there could be differences in the biodistribution of [^111^In]In-DOTA-trastuzumab and [^225^Ac]Ac-DOTA-trastuzumab. Nonetheless, the tumour uptake in NRG mice with s.c. 164/8-1B/H2N.luc + HER2-positive BC xenografts was > twofold significantly higher (*P* < 0.0001) in mice co-injected with [^111^In]In-DOTA-trastuzumab and [^225^Ac]Ac-DOTA-trastuzumab than in mice coinjected with [^111^In]In-DOTA-IgG_1_ and [^225^Ac]Ac-DOTA-IgG_1_ (10.6 ± 0.6% ID/g vs. 4.3 ± 0.7% ID/g, Fig. 5a), demonstrating HER2-specific tumour uptake. However, spleen uptake in mice coinjected with [^111^In]In-DOTA-trastuzumab and [^225^Ac]Ac-DOTA-trastuzumab was high (28.9 ± 7.4% ID/g) and was even higher in mice coinjected with [^111^In]In-DOTA-IgG_1_ and [^225^Ac]Ac-DOTA-IgG_1_ (100.6 ± 36.2% ID/g). Sharma et al. (2018) similarly reported high spleen uptake of i.v. injected ^89^Zr-labeled mAbs in immunocompromised strains of mice that do not have B-cells (e.g. scid, NOD scid and NSG), but not in athymic (nu/nu) mice which have B-cells but not T-cells. Notably, spleen uptake in NSG mice with s.c. SKOV-3 human ovarian cancer xenografts injected i.v. with 0.9–1.1 MBq (4.4–5.2 µg) of ^89^Zr-labeled trastuzumab was very high (> 300% ID/g) at 144 h p.i., causing low tumour uptake (~ 3% ID/g). This phenomenon was avoided by coinjecting ^89^Zr-labeled trastuzumab with an excess of isotype-matched IgG (220 µg). NRG mice have no B-cells and this may explain the high spleen uptake in mice coinjected with [^111^In]In-DOTA-trastuzumab and [^225^Ac]Ac-DOTA-trastuzumab. However, we injected a 8–10-fold higher total mass of RICs than reported by Sharma et al. (2018) (40 µg vs. 4.4–5.2 µg) which may have mitigated spleen sequestration resulting in higher tumour uptake in our study. The phenomenon of high spleen uptake of RICs may not occur in humans due to higher normal levels of circulating immunoglobulins. Nonetheless, high spleen sequestration of [^225^Ac]Ac-DOTA-trastuzumab may have toxicity implications for studying α-particle RIT in tumour-bearing NRG mice, Spleen toxicity may present in the form of reduced spleen size and weight, loss of integrity, orientation, or structure, and evidence of cellular necrosis (Cortez et al. 2020; Davis et al. 1999; Maguire et al. 2014). Increasing the administered mass of [^225^Ac]Ac-DOTA-trastuzumab may reduce spleen uptake and increase tumour uptake, minimizing toxicity and improving the response to RIT in preclinical studies. Tumours were well-visualized by SPECT/CT imaging in mice coinjected with [^111^In]In-DOTA-trastuzumab and [^225^Ac]Ac-DOTA-trastuzumab but not in mice coinjected with [^111^In]In-DOTA-IgG_1_ and [^225^Ac]Ac-DOTA-IgG_1_ (Fig. 5b). The ability to image tumours with [^111^In]In-DOTA-trastuzumab by SPECT/CT and treat tumours by RIT with [^225^Ac]Ac-DOTA-trastuzumab presents an opportunity for a theranostic strategy for imaging and treatment of HER2-positive BC. Moreover, since 164/8-1B/H2N.luc + tumours in immunocompromised mice metastasize to the brain (Milsom et al. 2013), these results support our planned future studies to examine the effectiveness of [^225^Ac]Ac-DOTA-trastuzumab for RIT of HER2-positive BM in this mouse tumour xenograft model.

## Conclusions

We conclude that [^225^Ac]Ac-DOTA-trastuzumab exhibited potent and HER2-specific cytotoxicity on SK-BR-3 cells in vitro and caused extensive DNA DSBs in the cell nucleus. Furthermore, [^225^Ac]Ac-DOTA-trastuzumab co-injected with [^111^In]In-DOTA-trastuzumab assuming that [^111^In]In-DOTA-trastuzumab radiotraces the uptake of [^225^Ac]Ac-DOTA-trastuzumab exhibited HER2-specific uptake in s.c. 164/8-1B/H2N.luc^+^ human BC tumours in NRG mice, allowing tumour imaging by SPECT/CT. These results are promising for combining [^111^In]In-DOTA-trastuzumab and [^225^Ac]Ac-DOTA-trastuzumab in a theranostic strategy for imaging and RIT of HER2-positive BC.

### Supplementary Information

**Additional file 1**. Supplementary figures and table.

## Data Availability

All data generated or analyzed during this study are included in this published article [and its supporting information files].

## Abbreviations

%IA(t): Percent incubated activity
%ID/g: Percent injected dose per gram
[^225^Ac]Ac(NO_3_)_3_: Actinium-225-nitrate
^111^InCl_3_: Indium-111-chloride
A_Cell_: Activity taken up by cell
Ã_Cell_: Time-integrated activity in cells
A_M_: Activity taken up by medium
Ã_M_: Time-integrated activity in the medium
Ã_ML_: Time-integrated activity in monolayer cells
AUC: Area under curve
BBB: Blood–brain-barrier
BC: Breast cancer
BM: Brain metastases
B_max_: Maximum number of binding sites per cell
CE: Conjugation efficiency
CT: Computed tomography
D: Mean radiation absorbed dose
D_10_: Dose required to decrease the surviving fraction of cells to 0.10
DCIS: Ductal carcinoma in situ
ddH_2_O: Double-distilled water
DOTA: 1,4,7,10-Tetraazacyclododecane-1,4,7,10-tetraacetic acid
DOTA-NHS: DOTA N-hydroxysuccinimide ester
DSB: Double-strand breaks
D_SF_: Doses in the cell during clonogenic survival assay
DTPA: Diethylenetriaminepentaacetic acid
D_γ-H2AX_: Doses in the cell during the γ-H2AX assay
FBS: Fetal bovine serum
HCl: Hydrochloric acid
HER2: Human epidermal growth factor receptor-2
i.v.: Intravenous
IA: Activity incubated with cells
IgG: Immunoglobulin G
ITLC-SG: Instant thin layer silica gel chromatography
k: Decay constant
K_D_: Dissociation constant
k_e_: Effective decay constant
LE: Labeling efficiency
LET: Linear energy transfer
mAb: Monoclonal antibody
MALDI-TOF MS: Matrix-assisted laser desorption ionization time-of-flight mass spectrometry
MCNP: Monte Carlo N-particle
MIRD: Medical Internal Radiation Dose
MU: Mass unit
MW: Molecular weight
NaOH: Sodium hydroxide
NLS: Nuclear translocation sequence
NRG: NOD-*Rag1*^*null*^* IL2rg*^*null*^
NSB: Non-specific binding
p.i.: Post-injection
PBS: Phosphate buffered saline
PE: Plating efficiency
PET: Positron-emission tomography
PSA: Prostate specific antigen
PSMA: Prostate specific membrane antigen
RBE: Relative biological effectiveness
RCP: Radiochemical purity
RIC: Radioimmunoconjugate
RIT: Radioimmunotherapy
ROS: Reactive oxygen species
RT: Room temperature
s.c.: Subcutaneous
SA: Specific activity
SB: Specific binding
S_C←C_: S values of cell to cell
S_C←CML_: S values of cell monolayer to cell
S_C←M_: S values of medium to cell
SD: Standard deviation
SDS-PAGE: Sodium dodecyl sulfate polyacrylamide gel electrophoresis
SE-HPLC: Size-exclusion high-performance liquid chromatography
SF: Surviving fraction
SPECT: Single photon emission computed tomography
T_b_: Biological half-life
TB: Total binding
T_e_: Effective half-life
T_p_: Physical half-life
t_R_: Retention time
γ-H2AX: Phosphorylated histone-2AX
