# Supplementary material for: [225Ac]Ac- and [111In]In-DOTA-trastuzumab theranostic pair: cellular dosimetry and cytotoxicity in vitro and tumour and normal tissue uptake in vivo in NRG mice with HER2-positive human breast cancer xenografts
Source: EJNMMI Radiopharm Chem. 2023 Sep 26;8:24. doi: 10.1186/s41181-023-00208-0 (PMC10522541; doi:10.1186/s41181-023-00208-0)
Supplement: Supplementary file 1 — Additional file 1. Supplementary figures and table. [file 41181_2023_208_MOESM1_ESM.docx]

**[^225^Ac]Ac- and [^111^In]In-DOTA-Trastuzumab Theranostic Pair – Cellular Dosimetry and Cytotoxicity *In Vitro* and Tumour and Normal Tissue Uptake *In Vivo* in NRG Mice with HER2-Positive Human Breast Cancer Xenografts**

Misaki Kondo^1^, Zhongli Cai^1^, Conrad Chan^1^, Nubaira Forkan^1^, Raymond M. Reilly^1,2,3,4^ *

^1^ Department of Pharmaceutical Sciences, University of Toronto, Toronto, ON, Canada

^2^ Princess Margaret Cancer Centre, Toronto, ON, Canada

^3^ Department of Medical Imaging, University of Toronto, Toronto, ON, Canada

^4^ Joint Department of Medical Imaging, University Health Network, Toronto, ON, Canada

**Supporting Information**

* Correspondence to: Raymond M Reilly; Email: [raymond.reilly@utoronto.ca](about:blank)

Leslie Dan Faculty of Pharmacy, University of Toronto, Toronto, ON, Canada M5S 3M2

Tel. 1-416-946-5522; FAX: 1-416-978-8511

**Fig. S1.** DOTA conjugated per molecule of trastuzumab determined by measuring the conjugation efficiency (CE) by ITLC-SG developed in 0.1 M sodium citrate buffer, pH 5.5 after trace labeling with ^111^In, then multiplying the CE by the molar excess of DOTA-NHS:trastuzumab in the conjugation reaction (10:1, 30:1 or 60:1). Conditions for trace labeling with ^111^In were: 0.1 M NH_4_ acetate buffer, pH 5.5 at 40 ^o^C for 30 min at a specific activity of 0.11 MBq/μg and trastuzumab concentration = 15 mg/mL. Values shown are the mean ± SD (n=3) of DOTA conjugated per trastuzumab molecule.

**Fig. S2.** Labeling efficiency (LE, %) of DOTA-trastuzumab with ^111^In in NH_4_Ac buffer, pH 5.5 under different conditions: **a.** incubation temperature [incubation time = 1.5 h and specific activity (SA) = 0.1 MBq/μg], **b.** incubation time (temperature = 40 ^o^C and SA = 0.1 MBq/μg), **c.** specific activity (SA; temperature = 40 ^o^C and time = 0.5 h), **d.** protein concentration (temperature = 40 ^o^C, time = 0.5 h and SA = 0.1 MBq/μg). Values shown are the mean ± SD labeling efficiency (n=3). There were no significant differences (*P*>0.05) in LE at the incubation temperatures, times, SA and protein concentrations studied.

**Fig. S3.** Labeling efficiency (LE) of DOTA-trastuzumab with ^111^In prepared using a 10:1, 30:1 or 60:1 molar excess of DOTA-NHS. Labeling conditions were: 1.5 h at 40 ^o^C in NH_4_Ac buffer, pH 5.5 at a SA=0.11 MBq/ug and protein concentration = 15 mg/mL. Values shown are the mean ± SD LE (n=3). Significant differences are indicated by the asterisk.

**Fig. S4.** Uptake of [^111^In]In-DOTA-trastuzumab (20 nmoles/L; 3.7 μg/mL) labeled at different SA (0.037-0.111 MBq/μg) by 1 × 10^5^ HER2-positive SK-BR-3 human BC cells in wells in 24-well plates incubated for 60, 120 or 180 mins at 37 ^o^C. Also shown is the cellular uptake of [^111^In]In-DOTA-trastuzumab (SA=0.074 MBq/μg) combined with a 100-fold excess of DOTA-trastuzumab to block HER2. Values shown are the mean ± SD (n=3).

**Table S1.** Time-integrated activity in HER2-positive SK-BR-3 cells incubated *in vitro* with 1.0 Bq of [^225^Ac]Ac-DOTA-trastuzumab labeled at different specific activities (SA)

| **SA** | **Ã_M,0-180min_ ^a^**  **(10^4^ Bq × s)** | **Ã_CML,0-180min_ ^a^**  **(Bq × s)** | **Ã_C180min-12d_ ^a^**  **(Bq × s)** |
| --- | --- | --- | --- |
| 0.037 MBq/μg | 1.032 ± 0.001 | 0.00199 ± 0.00004 | 0.032 ± 0.003 |
| 0.074 MBq/μg | 1.036 ± 0.001 | 0.00184 ± 0.00005 | 0.032 ± 0.004 |
| 0.111 MBq/μg | 1.036 ± 0.001 | 0.00183 ± 0.00003 | 0.035 ± 0.002 |
| 0.074 MBq/μg  + Blocking ^b^ | 1.075 ± 0.001 | 0.00003 ± 0.00001 | 0.0006 ± 0.0003 |

^a^ Ã_M,0-3h_: time-integrated activity in the medium above the monolayer cells over the 180 min treatment period in the clonogenic assay; Ã_CML,0-3h_: time-integrated activity per cell in the cell monolayer over the 180 min treatment period; Ã_C180min-12d_: time-integrated activity in an individual cell during the 12 d culture period after seeding treated cells in the clonogenic survival assay

^b^ Cells were treated with [^225^Ac]Ac-DOTA-trastuzumab combined with a 100-fold molar excess of DOTA-trastuzumab to block HER2
